# Reablement – relevant factors for implementation: an exploratory sequential mixed-methods study design

**DOI:** 10.1186/s12913-022-08355-x

**Published:** 2022-07-28

**Authors:** Theres Wess, Wolfgang Steiner, Mona Dür, Jessie Janssen

**Affiliations:** 1 Department of Physiotherapy, University of Applied Sciences, Favoritenstraße 226, 1100 Vienna, Austria; 2 Vienna Social Services, Vienna, Austria; 3 duervation, Krems, Austria; 4 Department of Health Sciences, IMC University of Applied Sciences Krems, Krems an der Donau, Austria

**Keywords:** Re-ablement, Restorative care, Rehabilitation at home, Elderly people, Mixed-methods-design, Community-dwelling older adults, Consolidated Framework of Implementation Research

## Abstract

**Background:**

Reablement is a multi-professional and internationally established home-based health care service for mainly older people with the aim to reduce the need for long-term care and to promote self-determination. However, it is unknown which factors would facilitate the implementation of reablement in health care services. Therefore, the aim of this work was to identify relevant factors for the implementation process and to elucidate their importance based on the perspectives of experts.

**Methods:**

Within an exploratory sequential mixed-methods design, a literature search followed by framework analysis was carried out using the five domains of the Consolidated Framework of Implementation Research (CFIR) to collect potentially relevant factors for implementation of reablement. A survey was then drawn up encompassing the factors identified. Within the survey international reablement – experts were asked to rate the relevance of these factors .

**Results:**

The literature search identified 58 publications that served as sources for the framework analysis, where 40 potentially relevant factors were clustered into the five CFIR domains. These 40 factors were rated by experts in an online-survey. Based on the analysis of survey-data, 35 factors were considered as relevant for implementation of reablement services. The CFIR-domain characteristics of individuals, including teamwork and communication skills, was seen as most relevant.

**Conclusions:**

The implementation of reablement services is complex and requires the consideration of numerous factors, especially regarding the CFIR-domain characteristics of individuals. From the perspective of the survey´s participants one important factor of a successful implementation was the engagement of the persons involved. It requires team members with a strong, shared vision. Communication skills are highly important to promote teamwork and intensive training is needed to establish these skills. Further research on the implementation of reablement services is essential to realize its full potential.

**Supplementary Information:**

The online version contains supplementary material available at 10.1186/s12913-022-08355-x.

## Background

Continuously increasing life expectancy and declining birth rates are leading to ageing societies in all Organisation for Economic Co-operation and Development (OECD) countries. Since 1970 the average life expectancy in OECD countries has increased by ten years [1]. This demographic change not only poses significant financial challenges for national budgets, but also entails social risks. Developments in age-related policies vary considerably from country to country. According to the Aging Society Index, Scandinavian countries and the USA, for example, are adapting better to the demands of an aging society than countries in Central, Southern and Eastern Europe [2].

Despite a lack of robust research [3], some authors indicate that reablement programs could be an effective instrument to meet the challenges of an aging society [4–10]. Starting in England in 2000, reablement was implemented in several OECD-countries´ health care services [8]. Reablement refers to a multi-professional, interdisciplinary service that aims to ensure or regain the greatest possible independence for the individual users—adults, predominantly older people, usually after discharge from inpatient treatment—enabling them to continue living as independently as possible in their own home [11].

The focus of this service is to help people to regain skills that are needed in daily life as well as on developing compensatory strategies to carry out everyday activities. For this purpose, classical aids and adaptations in the home environment as well as new technologies are used. The goal of reablement is to enable its users to fulfil their everyday needs as independently as possible, so that less or ideally no further care is necessary [9].

Even though the effectiveness of reablement services has been suggested, no such services have been implemented to date in Central, Southern and Eastern Europe [12, 13]. “Implementation is the process of putting to use or integrating evidence-based interventions within a setting" ^(^[14]^, p.118)^. However, the literature on the implementation of reablement services is rare.

Innovative solutions, especially in the form of complex interventions such as reablement services, are usually difficult to implement in daily practice [15]. Therefore it is necessary to focus on factors that are influencing implementation processes. Tabak et al. defined the Consolidated Framework of Implementation Research (CFIR) as a model that focusses exclusively on the integration of evidence-based interventions into practice [16]. This framework provides a comprehensive overview of all constructs that influence the process of implementation [17] and describes five domains. Those domains are *intervention characteristics*, *outer setting*, *inner setting*, *characteristics of individuals* and *process* [18]. According to Kirk et al. the use of the CFIR has been common since its development in 2009 and it is recommended to integrate the framework throughout the whole research and implementation process [19].

### Objective

Due to the lack of knowledge about which factors are relevant for the implementation of reablement services in the community, the following two questions are asked:

Which potentially relevant factors for the implementation of reablement can be identified in the current literature of internationally implemented reablement services?

How relevant are these factors for the implementation of reablement services based on the perspectives of international reablement experts?

Hence, the aim of this work was to identify relevant factors for the implementation process and how international experts prioritised these factors.

## Methods

To answer the research questions, an exploratory sequential mixed-methods design was used. This design usually occurs in three phases, in which the analysis of qualitative data is followed by a development phase of translating the qualitative findings into a questionnaire. In the final third phase, quantitative data is collected and analysed ^(^[20]^,p.84)^.

**Fig. 1:**
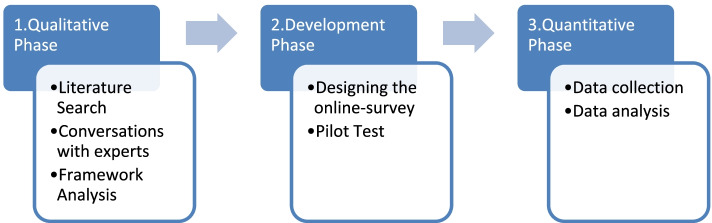
Exploratory sequential mixed-methods design

In this work potentially relevant factors were identified within a framework analysis based on a literature review and discussions with reablement experts (phase 1). In the second phase a survey was developed and piloted, whereas in the third phase the survey was distributed (see Fig. 1).

### Identifying potentially relevant factors for the implementation of reablement services

To identify potentially relevant factors for implementing reablement services a literature review and conversations with reablement experts formed the base for a framework analysis.

The literature search was conducted in the *Cochrane Library/Wiley*, *Pubmed/Medline*, and *Cinahl/Ebsco* databases. The following search terms in title or abstract were used: *reablement* OR *re-ablement* OR *restorative care*. A corresponding search was carried out for theses at libraries of German-speaking universities of applied sciences as well as an internet search for reference books on the subject of reablement. Subsequently, the bibliographies of all sources found in this process were reviewed. Two authors (TW & WS) read through the title and abstract of the publications listed, subsequently screened all abstracts on relevance, duplicates and year of publication. Publications prior to 2000 were excluded, as well as publications for which both researchers agreed consensually, after reviewing the abstract, that a content-related or subject-related reference to the research object was missing. Relevant publications were progressed to the full article stage.

In addition to the literature search, conversations were held with reablement experts in Finland. These experts were identified and invited to a conversation based on purposeful sampling. The interviewees selected were practitioners in reablement services in Finland. As reablement is a relatively young concept for Finland—implementation started in 2014—the findings from the implementation phase were still current and all interviewed practitioners remembered the process of implementation well. A total of four institutions with reablement services were visited in June 2019 in the municipalities of Helsinki, Lappeenranta, Mikkeli and Tampere. The conversations were conducted narratively, openly and unstructured, without a guideline; two authors (TW & WS) personally interviewed the experts and invited them to think aloud ^(^[21]^,p.358)^ starting with: “Can you tell us about your experience with and during the implementation of reablement in your organisation / community? What worked, why, what didn't?” The researchers took notes, which were then compiled into a memory protocol. In addition to the literature found, the protocol served as an additional source for the framework analysis.

Finally, the available full articles and the memory protocol were read and potentially relevant factors for the implementation of reablement programs were extracted with the use of framework analysis. No rigour quality assessment of the articles took place as the aim of the search was to identify as many factors as possible which were potentially relevant for implementation. The relevance of these factors would be decided by the reablement experts in phase 3. Framework analysis was developed by the National Centre for Social Research in the United Kingdom and is a more deductive form of analysis [22]. The domains of the CFIR were used as a deductive frame of reference to categorize the factors [17]. More than one factor could be extracted from every article and put in the relevant domain. Discrepancies between the two authors were discussed until consensus was reached.

### Designing the survey

Following the identification of potentially relevant factors, an online survey was designed. The list of potentially relevant factors served as items. A Likert scale ranging from 1 (not relevant at all) to 5 (highly relevant) was used to rate the relevance of the items. The technical tool used was a software called LimeSurvey [23].

The survey was tested on two people who had experience with reablement in a pre-test in order to identify and correct any difficulties the respondents might have with the inquiry. As the survey was designed in English, but was also intended to be answered by experts whose native language was not English, one non-native speaking expert, a Finish consultant for the implementation of reablement, and one native speaking expert, an US health economist who has published on the cost-effectiveness of reablement in England, were chosen for pre-testing the survey.

Afterwards, the feedback was incorporated so that a final version of the survey was available at the end of this phase.

### Collecting and analysing the survey-data

The survey-link was sent out via email to practitioners in reablement services and researchers with publication history on reablement and was live between October 2019 and January 2020. The snowball procedure, where every person contacted was pegged to forward the link, was chosen to obtain a non-probabilistic sample. The fact that this sampling procedure reduced the representativeness of the study results was accepted. Data was collected anonymously, then exported from LimeSurvey and imported to and analysed with IBM SPSS Statistics 26 and Excel 2016. Since the factors were evaluated using a five-point Likert scale, the median, minimum, maximum and quartiles were determined as statistical measures.

Ethical considerations: In the present work, the authors have followed the principles of the Good Reporting of a Mixed Methods Study (GRAMMS) [24]. The responsible ethics committee was asked for an assessment and decided that no ethics vote was necessary. All survey participants gave their informed consent.

## Results

### Potentially relevant factors for the implementation of reablement

In total 184 publications considering the implementation of reablement services were identified: 160 were found through the literature search at the selected databases and 24 were additionally included from the references of the publications as well as through an online search for reference books and theses.

**Fig. 2:**
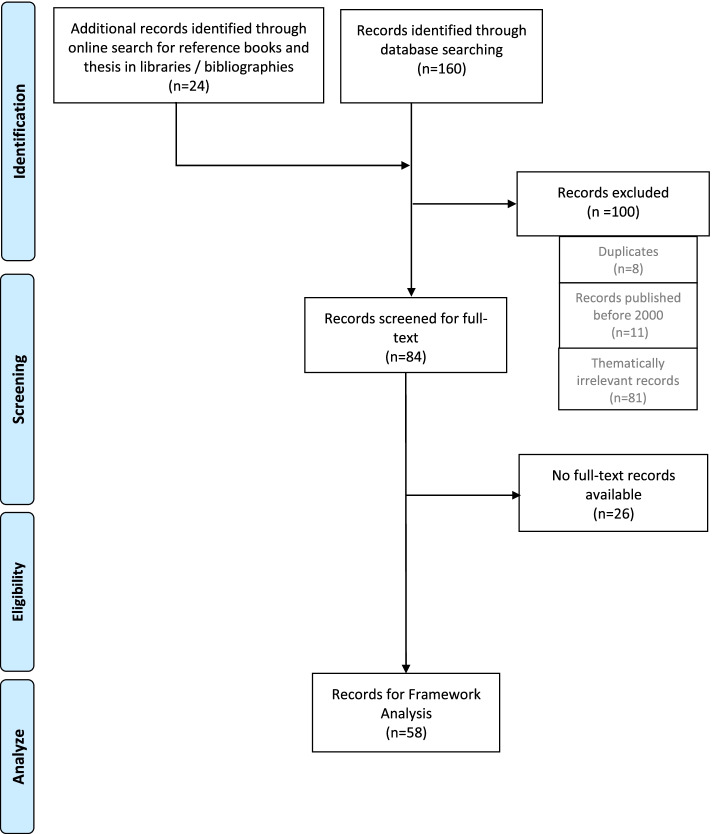
Literature search – PRISMA flowchart

Finally, 58 publications could be identified as relevant in terms of subject and topic and thus served as sources for the framework analysis. Figure 2 shows the PRISMA flowchart of the publications in the literature search.

In total, potentially relevant factors for the implementation of reablement were found in 15 of 58 sources. These 15 sources are listed in Table 1. Sources in which factors were only vaguely hinted at, but which are formulated much more clearly in other sources, were not included in the list. For example, in the qualitative study "The user voice: older people's experience of reablement and rehabilitation", generally formulated findings such as "The data showed that individuals need a range of interventions and techniques at different stages of recovery" ^(^[25]^, p.185)^ or "Whichever the setting, the key to rehabilitation appears to be the way staff motivate people to engage in it. Study participants described both "hands-on" and "hands-off" tactics" ^(^[25]^, p.187)^. Both the question of the composition of the team and service and the therapeutic and nursing techniques used are addressed more explicitly in other sources. Accordingly, the study was not included in the list of sources from which potentially relevant factors were extracted.

**Table 1:** Sources mentioning potentially relevant factors for the implementation of reablement

#	Authorship	Title	Publication type/Design
1	Lewin, Alfonso & Alan [4]	Evidence for the long term cost effectiveness of home care reablement programs	Quantitative study
2	Hjelle, Tuntland, Forland & Alvsvag. [26]	Driving forces for home-based reablement; a qualitative study of older adults ‘experiences	Qualitative study
3	Birkland, Tuntland, Forland, Jakobsen & Langeland. [27]	Interdisciplinary collaboration in reablement—a qualitative study	Qualitative study
4	Tessier, Beaulieu, McGinn & Latulippe [6]	Effectiveness of Reablement: A Systematic Review	Systematic literature review
5	Lewin, Concanen & Youens [28]	The Home Independence Program with non-health professionals as care managers: an evaluation	Quantitative study
6	Tinetti, Baker, Gallo, Nanda, Charpentier & O'Leary [12]	Evaluation of Restorative Care vs Usual Care for Older Adults Receiving an Acute Episode of Home Care	Quantitative study
7	Rabiee & Glendinning [29]	Organisation and delivery of home care re-ablement: what makes a difference?	Quantitative study
8	Moe & Brinchmann [30]	Tailoring reablement: A grounded theory study of establishing reablement in a community setting in Norway	Qualitative study
9	Eliassen, Henriksen & Moe [31]	The practice of support personnel, supervised by physiotherapists, in Norwegian reablement services	Qualitative study
10	Hjelle, Alvsvåg & Førland [32]	The reablement team’s voice: A qualitative study of how an integrated multidisciplinary team experiences participation in reablement	Qualitative study
11	Tinetti, Charpentier, Gottschalk & Baker [33]	Effect of a Restorative Model of Posthospital Home Care on Hospital Readmissions	Quantitative study
12	Randström, Wengler, Asplund & Svedlund [34]	Working with ‘hands-off’ support: a qualitative study of multidisciplinary teams’ experiences of home rehabilitation for older people	Qualitative study
13	Social Care Institute for Excellence [35]	Maximising the potential of reablement	Report
14	National Institute for Health and Care Excellence [36]	Guideline scope—Intermediate care, including reablement	Guideline
15	Ebrahimi, Chapman [37]	Reablement Services in Health and Social care	Reference book

The framework analysis of the literature and the memory protocol led to a total of 40 potentially relevant factors structured into the five CFIR-domains. These factors are listed in Table 2.

**Table 2:** Factors according to the CFIR domains

Code	Factor
**Intervention characteristics (IC)**	
IC 01	Social issues, the need of "being" and "belonging" in the community are addressed; Reablement may take place in the wider community, not only in people´s homes
IC 02	Social environment (family, friends, neighbours) is included in the Reablement-team
IC 03	Client´s own goals serve as a common interdisciplinary platform; there is an agreement on the process for reaching these goals with the client, family and informal carers
IC 04	Care professional and therapist together carry out initial goal setting interview with clients
IC 05	Common, thorough and consistent assessments and documentation, e.g., development and implementation of a unified treatment-plan
IC 06	Evaluation on the basis of outcome and goal attainment instead of evaluation on time and tasks
IC 07	Care workers are exclusively employed in Reablement, so they don´t have to switch between Reablement and traditional home care
IC 08	Physiotherapists are part of the core team
IC 09	Occupational Therapists are part of the core team
IC 10	Physicians (gerontologist) are part of the core team
IC 11	Access to specialists` skills (e.g., dietician, substance abuse counsellor, mental health nurse, speech therapist…) is assured
IC 12	Access to equipment (e.g. aids, assistive technology, home adaptation and telecare) is given
IC 13	Face to face contact is minimised (use of phone calls and telecare instead) to avoid the chance that clients will become dependent on team members` visits and to ensure the program is as cost efficient as possible
IC 14	Reablement service is cost-free for clients
**Outer setting (OS)**	
OS 01	Political pressure to develop cost-effective solutions in response to demographic developments
OS 02	There is peer-pressure: other home care services are providing Reablement
OS 03	A selective approach to the Reablement service is used (that means excluding people who are unlikely to benefit from Reablement)
OS 04	The client has few or no previous experiences with traditional homecare
OS 05	The client has realistic expectations based on an understanding of the difference between traditional home care und Reablement
OS 06	The client has capacity to consent and has rehabilitation potential (e.g. not requiring total assistance with care and not bedridden)
OS 07	The client has willpower and motivation to work with the Reablement team towards autonomy
OS 08	The client has sufficient language skills to be able to communicate in the local language
**Inner setting (IS)**	
IS 01	There is a commitment to the philosophy and concept of Reablement as well as the value of occupation in the whole organisation
IS 02	Within the Reablement-team everyone is more or less at the same hierarchical level
IS 03	There is an agreement on the process for reaching client-centred goals within the Reablement-team
IS 04	The service has the capacity to provide flexible and prompt interventions (e.g. flexible use of working hours, goal-orientated planning of visits in terms of duration and frequency, adjust intervention quickly in response to improvements in clients´ abilities)
IS 05	Subsequent services after Reablement are provided in a way that maintains any progress the client has made
**Characteristics of individuals (CI)**	
CI 01	Reablement team members share a strong vision of the service (shared understanding of the aims and objectives of Reablement, especially to prevent inappropriate referrals)
CI 02	Reablement team members have individual qualities and social skills to perform teamwork / multidisciplinary collaboration / learning from each other
CI 03	Reablement team members use patterns of communication that encourage clients and their families to participate in all care decisions (that means promoting their sense of autonomy rather than exerting power or control over the client)
CI 04	Careworkers are trained on the principles of delivering a Reablement service (e.g., learning to “stand back”, principles of self-management, healthy aging…)
CI 05	Careworkers work on reaching the goals without focus on time and task and plan the duration of home visits individually
CI 06	Staff has less experience in traditional home care and is able to adapt more easily to the Reablement approach
**Process (P)**	
P 01	Communities design their own individual model of Reablement (including the structure of the team)
P 02	Rehabilitation experts are included in the planning and implementation of Reablement
P 03	Reablement initiatives should be individually tailored and flexible with opportunities for employees to be creative
P 04	Start-up costs and training for home care workforce are planned and calculated
P 05	Reablement pilot projects start with small teams of selected, motivated team-members
P 06	Success stories of selected users are promoted within the team
P 07	A high level of work satisfaction among the team is secured by planning and conducting appropriate measures

### Survey development

The survey designed in the development phase was fully standardized and highly structured: each factor was rated by the individual participants on a five-point Likert Scale (ordinal scaling). The prompt was: "Please rate the following factors for their relevance to the implementation of reablement! 1 = not relevant at all, 2 = rather not relevant, 3 = moderately relevant, 4 = rather relevant, 5 = highly relevant." For each factor, "no answer" could also be selected. After each domain there was the possibility to leave a comment.

The feedback of the two pre-testers concerning processing time and comprehensibility was very positive. However, pre-testers suggested to change the order of the individual factors. Therefore, a topic-related clustering of the factors was carried out in the revision of the survey.

### Relevant factors for the implementation of reablement services

Sixteen experts, from eight countries and five different professions, completed the survey. Most of the participants (*n* = 6) had over 10 years of experience with reablement services. In Table 3 their demographic data is represented.

**Table 3:** Participants’ demographic data

	*n* = 16	%
**Gender**		
Male	1	6,25
Female	14	87,5
n.m	1	6,25
**Nationality**		
Australia	4	25
Finland	2	12,5
Norway	2	12,5
Sweden	1	6,25
UK	3	18,75
USA	2	12,5
Germany	1	6,25
Canada	1	6,25
**Profession (multiple answers possible)**		
Nurse	2	
Occupational Therapist	4	
Physiotherapist	1	
Researcher	10	
Others	3	
**Experiences with reablement (y)**		
0–2	2	12,5
2–4	2	12,5
4–6	3	18,75
6–10	1	6,25
> 10	6	37,5
n.m	1	6,25

*n.m.* not mentioned, *y* years

**Fig. 3:**
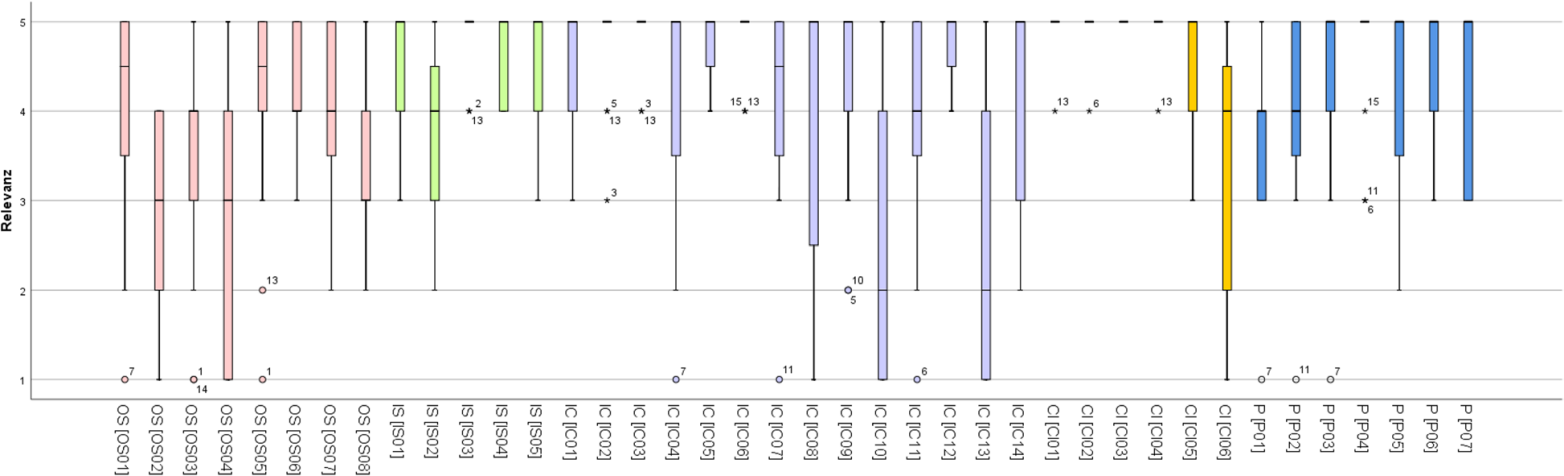
Relevant factors for the implementation of reablement services – box plots. Legend: OS- outer setting (in pink); IS- inner setting (in green); IC- intervention characteristics (in purple); CI – characteristics of individuals (in yellow); P- process (in blue); 1 = not relevant at all; 2 = rather not relevant; 3 = moderately relevant, 4 = rather relevant, 5 = highly relevant. Note: To read each factor in full text – see Table 2

The results for the 40 identified factors are illustrated in Fig. 3 by means of box plots. The results of the individual factors are arranged according to the survey and the five CFIR-domains.

Table 4 presents the mode, minimum, maximum and median for each factor.

**Table 4:** Relevant factors for the implementation of reablement services – listed results

factor	mode	minimum	maximum	median
OS 1	5	1	5	4,5
OS 2	4	1	4	3
OS 3	4	1	5	4
OS 4	1	1	5	3
OS 5	5	1	5	4,5
OS 6	4	3	5	4
OS 7	4	2	5	4
OS 8	3	2	5	3
IS 1	5	3	5	5
IS 2	4	2	5	4
IS 3	5	4	5	5
IS 4	5	4	5	5
IS 5	5	3	5	5
IC 1	5	3	5	5
IC 2	5	3	5	5
IC 3	5	4	5	5
IC 4	5	1	5	5
IC 5	5	4	5	5
IC 6	5	4	5	5
IC 7	5	1	5	4,5
IC 8	5	1	5	5
IC 9	5	2	5	5
IC 10	1	1	5	2
IC 11	5	1	5	4
IC 12	5	4	5	5
IC 13	1	1	5	2
IC 14	5	2	5	5
CI 1	5	4	5	5
CI 2	5	4	5	5
CI 3	5	5	5	5
CI 4	5	4	5	5
CI 5	5	3	5	5
CI 6	4	1	5	4
P 1	4	1	5	4
P 2	5	1	5	4
P 3	5	1	5	5
P 4	5	3	5	5
P 5	5	2	5	5
P 6	5	3	5	5
P 7	5	3	5	5

*OS* Outer setting, *IS* Inner setting, *IC* Intervention characteristics, *CI* Characteristics of individuals, *P* process

1 = not relevant at all; 2 = rather not relevant; 3 = moderately relevant, 4 = rather relevant, 5 = highly relevant

To read each factor in full text – see Table 2

Overall, most of the factors (24 out of 40) were rated with a median of five (highly relevant), while a further eleven had a median of at least four (rather relevant). Thus, 35 of the 40 listed potentially relevant factors were actually relevant or rather relevant.

Nine factors were rated as particularly highly: They had a median of five and not more than three experts rated them not as highly relevant. Table 5 shows these items, listed according to their respective domains in the CFIR. These nine most relevant factors were distributed over four out of the five CFIR domains. Four of the nine factors rated as particularly relevant were assigned to the domain *characteristics of individuals*. The domain *intervention characteristics* was represented by three factors, the domains *inner setting* and *process* by one factor.

**Table 5:** Most relevant factors for the implementation of reablement services

Code	Factor
IS 03	There is an agreement on the process for reaching client-centred goals within the reablement-team
IC 02	Social environment (family, friends, neighbours) is included in the reablement-team
IC 03	Client´s own goals serve as a common interdisciplinary platform; there is an agreement on the process for reaching these goals with the client, family and informal carers
IC 06	Evaluation on the basis of outcome and goal attainment instead of evaluation on time and tasks
CI 01	Reablement team members share a strong vision of the service (shared understanding of the aims and objectives of reablement, especially to prevent inappropriate referrals)
CI 02	Reablement team members have individual qualities and social skills to perform teamwork / multidisciplinary collaboration / learning from each other
CI 03	Reablement team members use patterns of communication that encourage clients and their families to participate in all care decisions (that means promoting their sense of autonomy rather than exerting power or control over the client)
CI 04	Careworkers are trained on the principles of delivering a reablement service (e.g., learning to “stand back”, principles of self-management, healthy aging…)
P 04	Start-up costs and training for home care workforce are planned and calculated

*IS* Inner setting, *IC* Intervention characteristics, *CI* Characteristics of individuals, *P* Process

Five factors were rated lower in relevance than the other 35. These five factors had a median of less than four. The factors "there is peer-pressure: other home care services are providing reablement" (OS02), "the client has few or no previous experiences with traditional homecare" (OS04) und "the client has sufficient language skills to be able to communicate in the local language" (OS08) were rated with a median of three, all three factors assigned to the domain *outer setting*. The factors “physicians (gerontologist) are part of the core team” (IC10) and “face to face contact is minimised (use of phone calls and telecare instead) to avoid the chance that clients will become dependent on team members´ visits and to ensure the program is as cost efficient as possible” (IC13) were rated with a median of two, both factors assigned to the domain *intervention characteristics*.

The evaluation of the comments given by the participants in the survey confirmed the relevance of the 40 extracted factors. There was no comment which indicated, that perceived important factors were missing in the survey. So the participants´ comments did not lead to new influencing factors.

## Discussion

This mixed-methods study directly addresses the implementation of reablement services. Previous publications that described, for example the “core characteristics of reablement” ^(^[6]^,p.51)^, drew their findings exclusively from literature research. In the present work, the results of the literature review were also verified by experts.

The selection of factors potentially relevant to the implementation of reablement proved to be comprehensive and precise. The survey´s participants largely confirmed the relevance of the selected factors. The idea that other relevant factors could have been missed during the identification process was opposed by the comments of the participants in the survey who did not provide any evidence that relevant factors were overseen.

The survey´s results showed that sixteen experts rated 35 out of 40 potentially relevant factors as relevant or rather relevant for the implementation of reablement. The high number of factors found to be relevant indicates that the process of implementing reablement is complex [15]. Respondents from different countries, with different basic professions and different backgrounds of experience took part in the survey. Fourteen of sixteen participants were female, which represents the distribution of females working in allied health professions [38].

The nine factors that emerged as most relevant were not evenly distributed across the five domains of the CFIR (see Table 4). Four of the nine factors were assigned to the domain *characteristics of individuals*, another three factors to the domain *intervention characteristics*. In the domain *characteristics of individuals* the patients’ and their carers´ goals were central, which reflects the philosophy of reablement [9]. This mirrors Safaeinili et al.‘s (2020) findings, who analysed 23 stakeholder transcripts with CFIR after a patient-centred intervention within a health setting, and found that patients and their needs were crucial for the successful development [39].

It can be said that all nine most relevant factors, even those that were not assigned to the domain *characteristics of individuals*, form a certain thematic unity concerning personal and social characteristics and behavioural aspects. The dominance of these aspects seems to be especially important. This reflects the statements of Valerie Ebrahimi and Hazel Chapman, who prominently stated that a paradigm shift is needed for the implementation of reablement: „ Importantly for reablement this involves a renegotiation of the values of health and social care support staff and professionals as well as those that use the service – a shift from ´doing to´ to ´doing with´ “ ^(^[37]^,p.47)^.

From the authors´ point of view, it is therefore permissible to prioritize the role of the people involved in the implementation of reablement as particularly relevant. This is in line with the finding of Damschroder et al. that implementation is primarily a social process ^(^[17]^,p.3)^.

Social skills and training of communication seemed to be especially important to the implementation process of reablement-programs. Therefore, a common vision, a common understanding of reablement is needed (see factor CI01). Team members must have special skills that promote teamwork. This is already referred to in a study by Moe, Ingstad and Brataas [40], who concluded that it is only through strong communicative skills that goal orientation and person-centeredness in reablement services can succeed. Intensive training is an important basis for acquiring these skills. This is also reflected in reablement training programmes, as an example, the *Brighton and Hove programme* had a strong focus on communication in its 2017 training materials ^(^[41]^,p.21)^. This idea was also supported by comments left by participants during the survey by scoring item P04: “Start-up costs and training for home care workforce are planned and calculated.” in the nine most relevant factors.

It was also stated as important that all participants commit to client-centred goal setting and that this goal is defined by everyone as a common platform. The evaluation of the reablement process should be done by checking the achievement of the goal, not only by the time spent. Furthermore, the social environment of the users should be included in the reablement team. To ensure that reablement is successfully implemented, a concrete cost plan must be prepared in advance, which also includes training costs. It seems to be advisable to start with a manageable pilot project covering a smaller region.

### Strengths and limitations

Reflecting on the whole research process, the authors can affirm that the use of a mixed-method design is particularly suitable due to the typical complexity of health care questions ^(^[24]^,p.169)^. The multi-professional composition of the research team allowed the research question to be addressed from the perspective of physiotherapy and occupational therapy.

Every step in the research process contributed to the following step: During the identification of potentially relevant factors, the addition of expert interviews aided the balanced representation of the CFIR-domains in the survey. Without this source, the memory protocol, the domain *process* (P) would have been underrepresented. This showed that the actual process of implementing reablement is described relatively little in the literature.

The number of answered questionnaires (*n* = 16) was low and affects the generalisability of the study greatly. It was assumed that a much higher response rate could be achieved by drawing a sample by means of the snowball method. Also renewed attempts by means of a reminder by mail did not increase the response. The reasons for the low response rate can only be speculated—the topic of reablement itself is usually met with a great response, and it is possible that so many questionnaire studies are currently being conducted that the respondents have become oversaturated. The generalisability of the study can therefore rightly be questioned as the results only represent the opinion of 16 respondents. However, the data collected was well distributed in terms of professions, nationality and professional experience.

The authors suggest repeating the survey in order to widen the number of involved institutions and completed data sets. Including the institutions and municipalities in which reablement services are implemented could enhance the number of participants. In addition, future research is needed to explore the user perspective in more depth. Another interesting aspect that may not have been highlighted enough is how societal factors contribute to the process. However, the results provide useful insight in key factors for the implementation of reablement services.

## Conclusions

The results of this study showed the following factors could contribute to a successful implementation of reablement services:

As reablement is a complex intervention, all key stakeholders should be involved in the implementation process and develop a common mind-set. This mind-set includes a clear commitment to client-centredness and multi-professional collaboration. Nurses of all qualification levels, occupational therapists, physiotherapists and case managers must be part of the reablement team. Funding, including all training processes, must be secured.

## Supplementary Information

**Additional file 1.**

**Additional file 2.**

## Data Availability

The anonymised datasets used and analysed during the current study are included in this published article and its supplementary information files.

## Abbreviations

CFIR: Consolidated Framework for Implementation Research
CI: Characteristics of Individuals
GRAMMS: Good Reporting of a Mixed Methods Study
IC: Intervention Characteristics
IS: Inner Setting
n.m.: Not mentioned
OS: Outer Setting
P: Process
PRISMA: Preferred Reporting Items for Systematic Reviews and Meta-Analyses
y: Years
