# Supplementary material for: Reablement – relevant factors for implementation: an exploratory sequential mixed-methods study design
Source: BMC Health Serv Res. 2022 Jul 28;22:959. doi: 10.1186/s12913-022-08355-x (PMC9336056; doi:10.1186/s12913-022-08355-x)
Supplement: Supplementary file 1 — Additional file 1. [file 12913_2022_8355_MOESM1_ESM.docx]

| **Domain „intervention characteristics“** | |
| --- | --- |
| Factor – Item in the survey | Text passages of the sources from which the respective factor was formulated |
| Social issues, the need of "being" and "belonging" in the community are addressed; Reablement may take place in the wider community, not only in people´s homes. | “the focus on what seems to be essentially daily living tasks, such as personal and domestic care, fails to address ´being´ and ´belonging´ in the community.” (15, p.93);  "Scope of services should address social needs" (4, p.55);  "reablement my take place in the wider community..." (13, p.3);  "getting support in one´s own home and social environment might be motivating for a larger number of older adults" (2, p.1588);  "experienced security and familiarity in their homes and community" (2, p.1587);  "opportunity to continue takin part in leisure activities and social life" (2, p.1585);  “an emphasis on capacity building or restorative care to maintain or promote clients … social participation.” (1, p.1274);  “they called for a greater focus on social and leisure activities and outdoor mobility.” (2, p.1582);  “…autonomy and connection to their social environment.” (2, p.1587); |
| Social environment (family, friends, neighbours) is included in the Reablement-team. | ”in addition, family, friends, neighbours and social environments were essential elements to regain confidence performing everyday activities.” (2, p.1587);  “Older person’s rehabilitation at home includes meeting different professions, involving family members and perhaps changing the dwelling based on the individuals’ physical abilities.” (12, p.30);  “family members were also seen to be sometimes resistant to re-ablement, preferring styles of intervention that minimised risk to older relatives. They wanted the reassurance of knowing that their elderly parents were being looked after, sometimes despite the wishes of the older person to retain their independence. Most front-line staff felt that explaining the aims of the re-ablement service to both service users and carers prior to their first visit could significantly help manage service users’ expectations and overcome informal carers’ perceptions of risk.” (7, p.500); |
| Care workers are exclusively employed in Reablement, so they don´t have to switch between Reablement and traditional home care. | “Staff in general and anecdotally home care support workers can find it difficult to switch between a traditional care role and a reablement role.” (15, p.86); |
| Client´s own goals serve as a common interdisciplinary platform; there is an agreement on the process for reaching these goals with the client, family and informal carers. | “participant´s own goals as a common interdisciplinary platform" (3, p.195);  "… a group of professionals, working together to achieve common goals" (3, S.196); "the goals were not defined by one of the professionals at the outset" (3, p.198);  "it´s not we who decide what to do, it is the patient´s own goals and they do not distinguish between professions" (3, p.198);  "the importance of shared goals in interdisciplinary collaboration is widely documented in various studies and must be seen as fundamental..." (3, p.200); "if a professional had defined the goals, the consequence might be a disagreement between the team members which consequently might lead to difficulties within the interdisciplinary collaboration" (3, p.200);  “the intervention must be person-centred and goal-directed.” (5, p.808);  “goal orientated care planning in partnership with client.” (5, p.809);  “Development and implementation of a unified plan of care based on goal attainment.” (11, p.1522); “agreement on the process for reaching client centred goals.” (11, p.1522); “goals based on input from the individual, family, and home care staff.”(11, p.1522);  “participants emphasised how they focused on older peoples resources and needs and were sensitive to their goals for rehabilitation.” (12, p.28); |
| Care workers work on reaching the goals without focus on time and task, and plan the duration of home visits individually. | “less focus on time and tasks; instead, reablement should be evaluated on the basis of the outcomes that the service will support the individual to achieve”(4, p.56);  “Reablement requires a move away from commissioning on the basis of time and tasks. Instead, reablement should be commissioned on the basis of the outcomes that the service will support the individual to achieve.” (13, p.1); |
| Evaluation on the basis of outcome and goal attainment instead of evaluation on time and tasks | “less focus on time and tasks; instead, reablement should be evaluated on the basis of the outcomes that the service will support the individual the achieve”(4, p.56);  “successful reablement services are outcome-focused with the express aim of improving the health and well-being of the people that need them.” (15, p.76);  “reablement requires a move away from commissioning on the basis of time and tasks. Instead, reablement should be commissioned on the basis of the outcomes that the service will support the individual to achieve.” (13, p.1);  “development and implementation of a uniﬁed plan of care based on goal attainment.” (11, p.1522); |
| Common, thorough and consistent assessments and documentation; e.g. development and implementation of a unified treatment-plan | "thorough and consistent recording system" (4, p.56);  “…systematic evaluation of needs and treatment plans for all.”(4, p.57);  “treatment plan reviewed regularly.”(4, p.51);  “the situation for participants in reablement is often complex, involving assessments from different professionals and close interdisciplinary collaboration.”(3, p.202);  “Ongoing assessment during the period of re-ablement was also important to enable the team to identify new targets as peoples abilities improved.” (7, p.499);  “barriers to collaboration include complex electronic medicals record and communication systems.” (8, p.117);  “development and implementation of a uniﬁed plan of care based on goal attainment.” (11, p.1522); |
| Physiotherapists are part of the core team. | “of particular importance was having quick access to OTs and physiotherapists.” (7, p.499); “physiotherapists are responsible for assessments, developing interventions and supervising home trainers, who mostly conduct the reablement training.” (9, p.108);  “participants highlighted the continuous supervision that was given by physiotherapists and occupational therapists. They felt that when physiotherapists and occupational therapists joined their teams, supportive rehabilitative approaches became more apparent and helped give person-centred rehabilitation.” (12, p.29);  “the overall skills mix of a reablement team is important. Although care workers are the foundation of a reablement service, social workers, nurses, physiotherapists and occupational therapists make important contributions and decisions need to be made about how to involve them.” (13, p.14); |
| Reablement service is cost-free for clients. | “some staff felt that service users’ resistance to re-ablement could be linked to the charges users thought they had to pay for the service, reporting consumerist attitudes of ´I pay your wages, you do that for me´.” (7, p.500); |
| Face to face contact is minimised (use of phone calls and telecare instead) to avoid the chance that clients will become dependent on team members` visits and to ensure the program is as cost efficient as possible. | “minimised face to face contact to avoid the chance that clients will become dependent on Team members visits and to ensure the program is as cost efficient as possible” (5, p.809) |
| Occupational Therapists are part of the core team. | “from a delivery perspective, it is argued that occupational therapists unique skills and training in rehabilitation, recovery and enabling, makes their involvement essential to the success of reablement services.” (15, p.79);  “A strong priority should be placed on the involvement of occupational therapy particularly in the planning of the service and supervision of reablement support staff.” (Skelton, 2013, zitiert nach 15, p.79);  “Having quick access to OTs to cope with demand was considered to be more important than having OTs necessarily embedded in the team.” (7, p.500);  “participants highlighted the continuous supervision that was given by physiotherapists and occupational therapists. They felt that when physiotherapists and occupational therapists joined their teams, supportive rehabilitative approaches became more apparent and helped give person-centred rehabilitation.” (12, p.29);  “the overall skills mix of a reablement team is important. Although care workers are the foundation of a reablement service, social workers, nurses, physiotherapists and occupational therapists make important contributions and decisions need to be made about how to involve them.” (13, p.14); |
| Access to specialists` skills (e.g. dietician, substance abuse counsellor, mental health nurse, speech therapist...) is assured. | “having access to a variety of other specialist skills in the team could enable the service to work more effectively with a wider range of user.” (7, p.500);  “having close relationships with and quick access to professionals and skills outside the re-ablement team was considered another factor making a huge difference to the type and quality of support the re-ablement team was able to offer.” (7, p.500);  “other professionals outside the team to whom quick access was said to be crucial included care management teams, hospital social work team, district nurses, continence advisors, community matrons and specialists for visually impaired clients.” (7, p.500);  “Professionals’ not necessarily full-time members of the team but frontline workers need access to specialist skills.”(4, p.56);  "addressing psychological support…is also vitally important" (4, p.56);  “enable team members to give better support to older peoples psychosocial needs.” (12, p.29); |
| Access to equipment (e.g. aids, assistive technology, home adaptation and telecare) is given. | “rapid provision of equipment such as grab rails or walking frames was considered a major part of re-ablement services.” (7, p.500);  "Access to equipment"(4, p.56); |

| **Domain „Outer Setting“** | |
| --- | --- |
| Factor – Item in the survey | Text passages of the sources from which the respective factor was formulated |
| The client has capacity to consent and has rehabilitation potential (e.g. not requiring total assistance with care and not bedridden). | "Participant´s ability to define their own goals therefore played a crucial role…" (3, p.200);  “With the hospital discharge model, it is likely that two of the criteria for referral to a reablement service is a health need, capacity to consent and rehabilitation potential.” (15, p.81);  “older persons at risk for functional decline after acute illness or hospitalization bit with the potential for maintaining or improving their function…not requiring total assistance with care and not bedridden.” (6, p.2099); |
| The client has few or no previous experiences with traditional homecare. | "reablement worked better for newly referred people" (4, p.56);  “a further complication in this debate is that recipients do not always comprehend fully what the aim or philosophy of reablement is. When this is the case, the outcome may be disappointment at not being ´looked after´. This is particularly relevant in the instances where formal care has been in place for many years.” (Glendinning, 2010, zitiert nach 15, p.50)  There was a consensus among front-line staff across all sites that re-ablement worked better for people newly referred to adult social care.” (7, p.500); |
| Political pressure to develop cost-effective solutions in response to demographic developments. | “We all have a cross-pressure between quality and budgets. We must try to balance and to maintain quality.” (8, p.118);  “Pressure on managers to demonstrate rapid improvements in functioning were highlighted as the key drivers to be selective.” (7, p.498);  “The anticipated shortage of health and care personnel and the increase in financial pressures have led to concerns about the sustainability of existing services.” (8, p.114);  “An increasing number of older adults and a growing demand for complex services have placed pressure on local governments to offer proactive interventions to strengthen citizens’ ability to remain independent in their home environment.” (9, p.108); |
| A selective approach to the Reablement service is used (that means excluding people who are unlikely to benefit from Reablement). | “People who are unlikely to benefit from this service are screened out.” (15, p.85);  “Participants felt that some older persons lacked motivation, owing to feelings of loneliness and poor physical health. At the same time, they said that home rehabilitation is not suitable for all older people, because not everyone feels secure in their homes when their health deteriorates” (12, p.28);  “in almost all settings, reablement is available to who need homecare services without discrimination, including those with cognitive impairment, for whom the evidence actually suggests less benefit.” (4, p.55);  "benefit may be less for those likely to need ongoing support such as people with dementia or mental health problems" (4, p.56);  “people should not be excluded from reablement on the basis of a dementia diagnosis. They should be assessed on the basis of their needs and strengths without prejudice about their potential to be ´reabled´.” (13, p.27); |
| The client has willpower and motivation to work with the Reablement team towards autonomy. | “The referral criteria for this kind of service … understanding of the concept of reablement and motivation to accept and work with the reablement team towards autonomy.” (15, p.81);  “my willpower is needed.” (2, p.1581);  “the willpower is perceived as important to exercise and performing everyday activities to achieve the goal for reablement.” (2, p.1585);  “participants in this study described how reablement…enabled them…to have autonomy.” (2, p.1585); |
| The client has realistic expectations based on an understanding of the difference between traditional home care und Reablement. | “the person’s motivation alongside realistic expectation are essential. They therefore need to be able to understand the difference between traditional home care and reablement.” (15, p.82);  “a further complication in this debate is that recipients do not always comprehend fully what the aim or philosophy of reablement is. When this is the case the outcome may be disappointment at not being ´looked after´” (Glendinning, 2010, zitiert nach 15, p.50);  “the aim of reablement is not to do things for people, or to provide assistance, as in traditional homecare, but to show people how they can do things for themselves.” (Wood & Salter, 2012, zitiert nach 15, p.77);  “previous receipt of conventional home-care services could create unhelpful expectations and resistance to change.” (7, p.500); |

| **Domain „Inner Setting“** | |
| --- | --- |
| Factor – Item in the survey | Text passages of the sources from which the respective factor was formulated |
| There is a commitment to the philosophy and concept of Reablement as well as the value of occupation in the whole organisation. | “…it remains undisputed that: the vehicle for change in reablement is through occupation.” (15, p.50);  “staff commitment, attitude and skills.” (4, p.56);  “… understanding of the concept of reablement and motivation to accept and work with the reablement team towards autonomy.” (15, p.81); |
| Within the Reablement-team, everyone is more or less at the same hierarchical level. | “The establishment of an interdisciplinary team where everyone was more or less at the same hierarchical level, resulted in the professionals being ‘forced’ to adhere to each other.” (3, p.198);  "… a group of professionals, ideally at the same hierarchical level, working together to achieve common goals" (3, p.196);  “…positive collaboration are most likely a result of the organization where professionals are put together and work side by side linked to embracing common goals.”(3, p.201);  "we are supporting each other in helping persons reach their own goals" (3, p.198);  “Reorganization of the home care staff from individual care providers into an integrated, interdisciplinary team with shared goal”.(11, p.1522); |
| The service has the capacity to provide flexible and prompt interventions (e.g. flexible use of working hours, goal-orientated planning of visits in terms of duration and frequency, adjust intervention quickly in response to improvements in clients´ abilities). | "flexible and prompt intervention" (4, p.51);  “an attempt to provide more timely, flexible, and targeted service that are capable of maximizing clients independence.” (1, p.1274);  “The importance of flexibility over the timing, duration of visits, the content of home visits and the ability to adjust the service quickly in response to improvements in users’ abilities, was highlighted by all participants.” “In sites […] where re-ablement was a specialised service, most front-line staff reported having greater flexibility in their re-ablement work compared with traditional home-care services. In contrast, in the site where re-ablement was part of the in-house service […] although re-ablement interventions took longer, they were allocated the same length of time for visits as conventional care. This suggests that staff in that site were more likely to take a conventional approach which took less time.” (7, p.499); |
| There is an agreement on the process for reaching client-centred goals within the Reablement-team. | “Establishment of goals based on input from the individual, family, and home care staff; agreement on the process for reaching these goals” (11, p.1522);  "professionals are put together and work side by side linked to embracing common goals" (3, p.201);  “…participants were enabled to define activity goals without restrictions and this was a major contribution to the willpower and intrinsic motivation.” (2, p.1587)  “During the initial COPM interview, the participants motivation was stimulated through defining own goals…their motivation was further enhanced due to the professional staffs support and supervision.” (2, p.1587); |
| Subsequent services after Reablement are provided in a way that maintains any progress the client has made. | “Limitations the reablement…include: Problems when care is transferred back to ´conventional´ home care workforce” (15, p.80);  “there was a widespread concern among most managers that as soon as service users were transferred, long-term care services could ´undo´ the work of re-ablement by doing things for people again.” (7, p.501);  “if a person has ongoing support needs at the end of reablement, it is crucial that subsequent services continue to provide support in a way that maintains any progress that a person had made.” (13, p.1); |

| **Domain „Characteristics of Individuals“** | |
| --- | --- |
| Factor – Item in the survey | Text passages of the sources from which the respective factor was formulated |
| Reablement team members share a strong vision of the service (shared understanding of the aims and objectives of Reablement, especially to prevent inappropriate referrals) | "strong and shared vision of the service" (4, p.56);  “…several professionals were fully dedicated to and shared a common focus on reablement.”(3, p.201);  “reorganization of the home care staff from individual care providers into an integrated, interdisciplinary team with shared goals.” (11, p.1522);  “an important success factor for re-ablement services was thought to be the extent to which everyone (…) had a shared understanding of the aims and objectives of the service.” (7, p.500);  “the fact that about 10 percentage of people returned to hospital at the end of the re-ablement period indicated the inappropriate nature of some referrals for re-ablement.” (7, p.500);  “many workers referred to their job as ´standing and watching´, but it was not clear whether they all appreciated that the observation was part of an ongoing assessment process and an important job in itself.” (7, p.499);  “from the managers’ point of view, one of the consequences of retraining the former home-care workforce was resistance from some staff who were not receptive to the new way of working.” (7, p.499); |
| Reablement team members have individual qualities and social skills to perform teamwork / multidisciplinary collaboration / learning from each other. | "learning from each other´s competencies" (3, p.195);  "staff commitment, attitude and skills" (4, p.56);  "new roles and joint efforts but specific competencies" (3, p.195);  “participiants said that ´working across professional boundaries was new to them, as they were used to working on their own professional capacity. Working with team members, and having a mutual understanding of other professions were described as stimulating. To further cooperation, participants described how essential it was to discuss each professions responsibilities.” (12, p.28); |
| Reablement team members use patterns of communication that encourage clients and their families to participate in all care decisions (that means promoting their sense of autonomy rather than exerting power or control over the client). | “use of language and patterns of communication that encourage clients and their families to participate in all care decisions and which promote their sense of autonomy rather than exerting power or control over the client”. (5,pS.809) |
| Caseworkers are trained on the principles of delivering a Reablement service (e.g. learning to “stand back”, principles of self-management, healthy aging...). | "Training on the principles of delivering a reablement service (e.g., learning to "stand back)" (4, p.56);  “reablement workers must learn to stand back and encourage people to regain or re-learn the ability to do things for themselves.“ (13, p.1);  “there is however, consensus that reablement is not effective unless care workers undergo specific training to understand the principles of delivering a reablement service.” (13, p.17);  “importantly for reablement this involves a renegotiation of the values of health and social care support staff and professionals as well as those that use services – a shift from ´doing to´ to ´doing with´. (Dewing, 2004, zitiert nach 15, p.47);  “the paradigm shift mentioned in earlier discussion – from ´hands-on” to ´hands-off”. (15, p.50);  “Reablement works may deliberately ´stand back´ and offer encouragement without actively assisting people in carrying out daily task.” (Wood & Salter, 2012, zitiert nach 15, p.77);  “All participants emphasised the importance of initial re-ablement training as well as ongoing training, team meetings and supervision to reinforce the re-ablement approach in day-to-day practice.” (7, p.499);  “reorientation of the focus of the home care team from primarily treating diseases and ´taking care of´ patients toward maximizing self-care function.” (11, p.1522);  “…focused on individual capabilities and resources and not ´doing´ too much, that is, providing ´hands off´ support.” (12, p.28); |
| Staff has less experience in traditional home care and is able to adapt more easily to the Reablement approach. | “”…staff with less experience in traditional home care were able to adapt more easily to this type of work. (15, p.86);  “most managers felt that staff with less experience of working in traditional home-care services were easier to train and had found the new approach less challenging.” (7, p.499);  “Limitations the reablement…include: issues with retraining the whole home care workforce” (15, p.80);  “from the managers point of view, one of the consequences of retraining the former home-care workforce was resistance from some staff who were not receptive to the new way of working.” (7, p.499); |

| **Domain „Process“** | |
| --- | --- |
| Factor – Item in the survey | Text passages of the sources from which the respective factor was formulated |
| Communities design their own individual model of Reablement (including the structure of the team). | “the usual challenges with establishing new services from other settings are mismatches between the characteristics of the new population, the local community and the original programme.” (8, p.114);  “adapting explains both adapting the reablement service to local conditions and adapting the community to this new way of offering and receiving public health services.” (8, p.117); |
| Reablement initiatives should be individually tailored and flexible with opportunities for employees to be creative. | “Reablement must be tailored to the existing services, and the geographic and demographic conditions.” (8, p.114);  “tailoring reablement also requires flexibility and professional autonomy.” (8, p.113);  “reablement is tailored when practitioners have the awareness to make individual and situational assessments.” (8, p.119);  “the service must be adapted to the local environment and community.” (10, p.583); |
| Start-up costs and training for home care workforce are planned and calculated. | “Limitations the reablement…include: start-up costs and issues with retraining the whole home care workforce” (15, p.80); |

Memory protokoll

| **factor** | **passage** | **domain** |  |
| --- | --- | --- | --- |
| Care professional and therapist together carry out initial goal setting interview with clients. | “initial interview with clients carried out by care professional and therapist together” (line 34) | CI |  |
| Physicians (gerontologist) are part of the core team. | “Doctors should be geriatric specialised and have a holistic view!” (line 241) | CI |  |
| There is peer-pressure: other home care services are providing Reablement. | “There is peer-pressure: other home care services are providing Reablement” (lines 62-63) | OS |  |
| The client has sufficient language skills to be able to communicate in the local language. | “sufficient knowledge of the language to be able to communicate in the national language” (line 179-180) | OS |  |
| There is a commitment to the philosophy and concept of Reablement as well as the value of occupation in the whole organisation. | “it´s a question of mindset, it needs a lot of education and reflexion going on and on; changing attitudes and values is hard to do.” (line 112-114) | IS |  |
| Success stories of selected users are promoted within the team. | “promoting success stories in the team” (line 40) | P |  |
| Rehabilitation experts are included in the planning and implementation of Reablement. | “rehabilitation experts included in the planning of reablement” (line 30) | P | |
| A high level of work satisfaction among the team is secured by planning and conducting appropriate measures. | “Planning and conducting measures to secure a high level of work satisfaction among the team” (line 33) | P | |
| Reablement pilot projects start with small teams of selected, motivated team-members. | “start pilot project with small team of selected, motivated team members” (lines 31-32) | P | |
| Care workers work on reaching the goals without focus on time and task and plan the duration of home visits individually. | “there should be no time-pressure on care workers” (line 37) | IC | |
